# A System for Traffic Violation Detection

**DOI:** 10.3390/s141122113

**Published:** 2014-11-24

**Authors:** Nourdine Aliane, Javier Fernandez, Mario Mata, Sergio Bemposta

**Affiliations:** Universidad Europea de Madrid, C/Tajo S/N, Urb el Bosque, Villaviciosa de Odón, Madrid 28670, Spain; E-Mails: javier.fernandez@uem.es (J.F.); mario.mata@uem.es (M.M.); sergio.bemposta@uem.es (S.B.)

**Keywords:** advanced driver assistance system, traffic sign detection, traffic, violation, safety, GPS, digital maps

## Abstract

This paper describes the framework and components of an experimental platform for an advanced driver assistance system (ADAS) aimed at providing drivers with a feedback about traffic violations they have committed during their driving. The system is able to detect some specific traffic violations, record data associated to these faults in a local data-base, and also allow visualization of the spatial and temporal information of these traffic violations in a geographical map using the standard Google Earth tool. The test-bed is mainly composed of two parts: a computer vision subsystem for traffic sign detection and recognition which operates during both day and nighttime, and an event data recorder (EDR) for recording data related to some specific traffic violations. The paper covers firstly the description of the hardware architecture and then presents the policies used for handling traffic violations.

## 1. Introduction

Road safety is one of the major subjects within the transport policy of the European Union Commission. In 2012 around 28,000 people were killed and more than 1,400,000 million injured in about 1,100,000 million traffic accidents on roads in the European Union [1]. Most traffic accidents are caused by driver inattention [2,3], distraction due to in-vehicle activities and fatigue [4,5]. In this aspect, drivers are not fully aware of their inattention or the distracting effects of in-vehicle tasks on their driving performance. In this context, the majority of traffic violations, such as speeding or ignoring stop signs, are unintentional and they occur due to a lack of concentration rather than because drivers deliberately intend to break the law. Thus, driving assistance systems for alerting drivers about their negligent behavior on the road and warning them to be more vigilant should be considered a primary solution for preventing accidents. It is important to stress that a device that can warn drivers about the speed limit at sites where driving at the wrong speed may result in an accident is a fundamental safety solution. These warnings should be issued with sufficient notice so that the driver has enough time to react to the oncoming traffic situation. Nevertheless, proper driving relies on drivers' accurate visual information and their appropriate reactions. In this aspect, traffic sign detection and recognition is critical for aiding drivers to understand the road conditions. This situation is particularly severe driving during nighttime. In this aspect, the use of computer vision is a valuable instrument to implement such systems, and some realizations can be found in [6–11]. It is worth it to mention that new advanced driver assistance system (ADAS) support drivers also in hazardous situations by braking [12]. However, there are situations where it is too late and full breaking will not avoid collisions, and ADAS provide steering intervention [13–15] as an additional option to prevent accidents.

Another approach to enhance safety is based on improving driver behavior, which may be achieved by a device able to monitor the driving [16–21]. In a recent survey on driving style [22], drivers were asked about a number of risky behaviors they might had had during the last year, and the most prevalent of these behaviors reported were speeding (89%) and driving when tired (56%) among other issues. Moreover, a research carried out in [23] shows that drivers involved in serious accidents where they are at fault have a personal traffic violations record with a number of faults clearly above the average. Thus, it seems that drivers with a record of several traffic violations are much likely to become involved in traffic accidents. Therefore, a system for recording and reporting traffic violations can be considered as an alternative for deterring drivers from their aggressive driving rather than conventional punishments.

To enhance the driving, and thereby safety, our approach consists in using a driver assistance system based on informing drivers about the traffic violations and faults they have committed. This approach may contribute to help drivers become more aware about their driving attitude and may persuade them to change their driving styles and, therefore, prevent them from committing unnecessary infringements. According to [24], traffic signs in front of vehicles often may not be seen by drivers, because of their different perceptions and lack of self-consciousness. Hence, traffic sign detection and identification is crucial to remind drivers about the existence of these signs, such as give way/yield, stops, speed limits and so forth. Perceiving traffic signs helps drivers to react accordingly in hazardous conditions. A number of methods and approaches have been developed for road sign detection and identification and some of them are reported in [24–28]. Readers can also find a general survey on this topic in [29].

Apart from alerting drivers about the traffic signs, it is also worthwhile to record the traffic violations and present drivers with feedback about their driving. In this aspect, this paper describes an experimental system aimed at detecting some traffic violations and then providing drivers with such feedback about their driving. The system is mainly based on a computer vision subsystem focused on traffic sign detection, and an event data recorder (EDR) unit for managing drivers' traffic violations.

The rest of the paper is organized as follows: first, the system's hardware architecture is described. Afterwards, the framework used for traffic violations detection and management is presented. In the following section, some preliminary results are discussed. Finally, some conclusions are drawn.

## 2. System Overview

**Figure 1.**
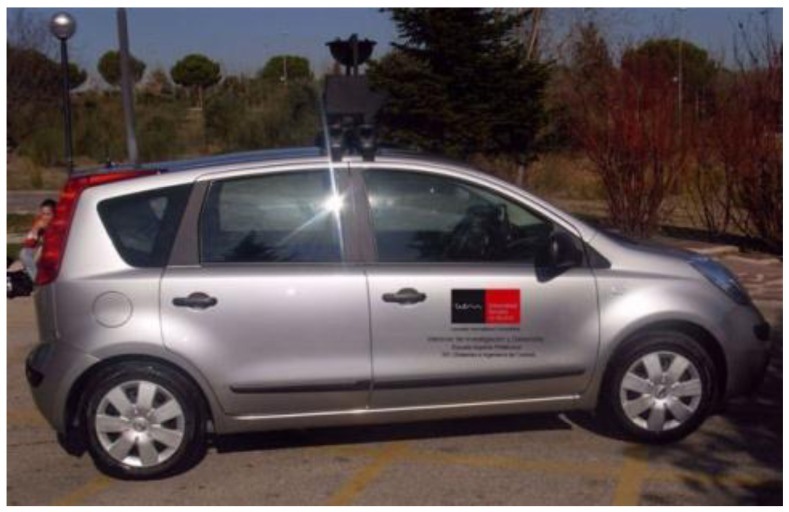
The experimental platform.

Developing a test-bed able to capture the vehicle surrounding and its internal state is not an easy task given the variety of available hardware and software choices. Besides being affordable, the different alternatives create a challenge for selecting the right equipment. The experimental platform is based on a conventional Nissan Note model as shown in Figure 1.

**Figure 2.**
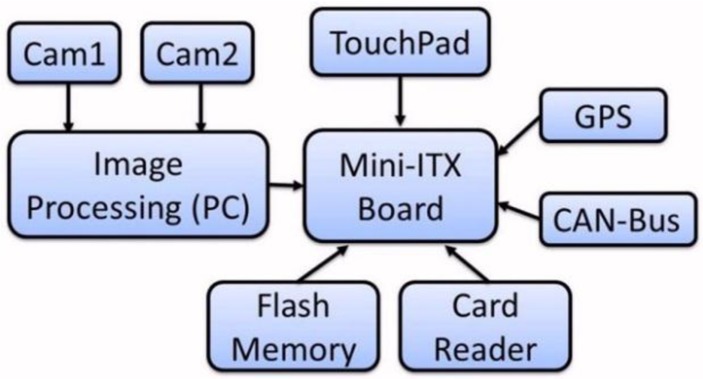
Hardware architecture.

Its embedded hardware consists of a Mini-ITX board as a host computer and a conventional PC as a slave computer for real-time image processing. Both of them are placed in a single rack located in the vehicle's boot. The Mini-ITX board integrates various readily available chipsets, such as a memory card adapter, a slot for connecting smart cards reader, a Bluetooth adaptor, *etc.* It also integrates some external devices, such as a GPS unit, a CAN interface, and a touchpad screen which permits access to system information as well as performing administrative tasks through an interactive GUI. All the electronic devices are powered by a dc/ac inverter, converting 12 Vdc from the vehicle battery to a 200 Vac, 600 W. The hardware architecture, as shown in Figure 2, is designed with the purpose of making the managing the distributed components with easy reconfiguration.

### 2.1. Vision Subsystem

The vision subsystem is equipped with two digital color cameras mounted on the vehicle's roof used to scan the road ahead looking for traffic signs. One camera is dedicated to daytime vision while the other is for night vision. Both cameras have a resolution of 1392 × 1040 and a focal length of 12 mm. This focal length has proven to be a good compromise between detection range (up to 80 m) and horizontal field of view (about 46°), retaining a good chance to pick close signs located at both sides of the vehicle. Using a 15 mm optic would obviously increase the detection range, but the narrower field of view could miss signs close to the vehicle in curves or roundabouts. On the other hand, using an 8 mm optic yields too short a detection distance for the system frame rate.

As far as night vision is concerned, the test-bed is equipped with an active near-infrared (NIR) source of illumination obtained with a regular headlamp with an infrared pass filter. The camera devoted to nighttime is a conventional one, but with its infrared filter removed in order to maximize the NIR light into the camera sensor, allowing the use of very short integration times. This fact helps to avoid motion blur and saturation of reflecting signs, and to remove non-reflecting objects.

### 2.2. Data Acquisition Subsystem

Some of the vehicle's internal data, such as rpm, acceleration, speed, and so on, is accessed through the vehicle ODB-CAN interface. This interface is normally used with specific scan tools for diagnosis issues and the ODB data transfer protocol follows several standards, none of which are directly compatible with PCs. This situation is solved by using the ELM327 IC as a bridge between the ODB port and the standard RS-232 interface. With this device, the raw data from the vehicle's ECUs is translated into short messages that are transmitted to a PC via a standard RS-232 connection. From a programming point of view, the ELM327 device is viewed as a modem supporting AT commands. Thus, from a standalone application, the vehicle's data is accessed using techniques emulating a conventional terminal and by issuing corresponding AT commands to a serial port. Other data, not supplied by the vehicle's ECU, are obtained by external instrumentation. For example, to measure the steering wheel position, a 7-bits absolute encoder is mounted on the steering shaft. The action of turning the steering wheel is then transformed into wheel angle information with respect to the neutral position. The full range of steering wheel is about three complete revolutions (−540° to 540°), so the encoder offers a resolution of 8.5° of steering wheel per bit, that is, approximately 1.2° wheel angle per bit. The Mini-ITX host is also connected to a GPS receiver with an update rate of 1 Hz, which is generally enough for automotive navigation. Finally, the system is equipped with several contact sensors to record driver interactions with the vehicle pedals, seat belts, indicators lights levers, and some other devices.

### 2.3. Smart Cards and User Profiles

Driver identification is carried out through the use of smart cards, and three types of users are implemented within the experimental platform: namely common drivers as system end-users, transport company agents acting as system administrators, and traffic enforcement agent users, which is reserved for future use. The different users are granted specific permissions regarding the use of different system functionalities. For example, a driver can only retrieve his own recorded data and his own traffic violations and is not allowed to retrieve data of other drivers. A transport company agent has access to the different functions, but he cannot modify stored data. If during a journey no smart card is used, the associated events are recorded as an anonymous driver account.

### 2.4. Event Data Recorder

The event data recorder (EDR) implemented in our test-bed uses two types of memory support. On the one hand a compact-flash memory, plugged directly into the Mini-ITX, is used to save the vehicle's internal data. In the other hand an external hard disk is used to store pictures and images related to detected traffic violations. Data is collected with approximately a rate of four records per second, and they are time-stamped and organized according to drivers. Besides vehicle internal data, special attention is given to traffic violations when they take place. In such situations, the identification of the traffic sign, its GPS location as well as a picture of the surrounding are recorded. The data acquisition process has a parallel nature with the constraint of sequential data-writing in the EDR unit (*i.e.*, no more than one event can be written at once), since the amount of data and the frequency at which they are acquired vary from one device to another. In this aspect, the adopted approach consists in organizing the program into multiple threads collecting data from different devices and a single thread does the ultimate writing to the EDR unit. Even though the purpose of the EDR is to provide drivers with feedback about their traffic violation records, it can be used for other tasks, for example for analyzing movement patterns, journey times, for acquiring more specific drivers' parameters, studies of why drivers make some decisions in certain scenarios, and thereby by use it for training and prevention.

### 2.5. Human-Machine Interface

**Figure 3.**
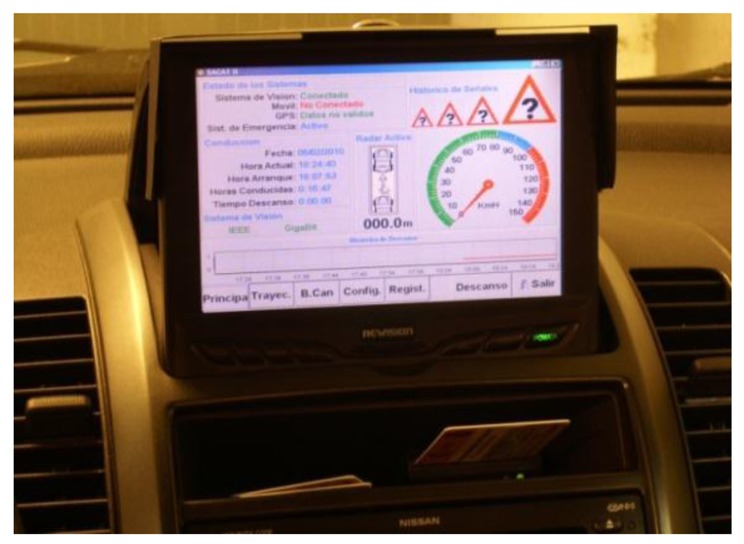
Tactile screen on the vehicle dashboard.

The test-bed is equipped with a tactile screen located on the dashboard running an HMI application as shown in Figure 3.

**Figure 4.**
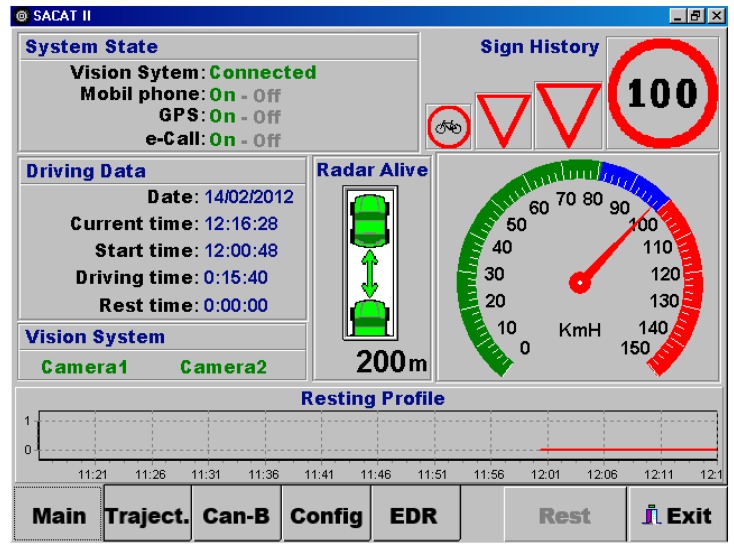
A snapshot of the HMI software application.

The front-end of the HMI is organized in multiple tabs giving users direct access to the different functionalities. The last four detected traffic signs are displayed on the main HMI screen. In the case of a traffic violation, a warning is emitted in the form of acoustical messages through the vehicle loudspeakers. Finally, drivers can also selectively activate or deactivate some of functionalities. Figure 4 shows a snapshot of the HMI software application.

## 3. Traffic Violations Detection and Management

In this section the test-bed framework for detecting some traffic violations and how they are reported to drivers is discussed. Firstly, the traffic sign detection and recognition (TSDR) subsystem is presented. Afterwards, the traffic violations recording unit (TVR) as well as the policies used for handling some traffic violations are discussed.

### 3.1. Traffic Sign Detection and Recognition

The traffic sign detection and recognition (TSDR) module, operating during both day and nighttime, is able to detect vertical signs present along the road. This module is aimed at alerting drivers in certain dangerous situations, such as “speeding”, “no passing zone”, “intersections”, “stop signs”, “yield/give way signs”, “dangerous turns”, “steep slopes”, or “road works”. In such situations, the system emits warnings in the form of acoustical messages through the vehicle loudspeakers. These warnings are issued with sufficient time to provide the driver with enough notice to react to the on-coming traffic situation.

The approach for the detection and recognition of traffic signs used in this work consists in combining several classical methods that solve different issues related to driving tests under uncontrolled lighting situations [24–29]. In the following section are presented the steps stages for traffic signs recognition during day and nighttime.

During daytime, the main stages are:

- Firstly, the camera integration time is adjusted dynamically in order to enhance the illumination of those image regions where typically signs appear. Otherwise, different illumination conditions will make this task impossible. Statistics are extracted on those image regions and averaged over the last shots to estimate the right shutter time for the next shot.
- Then, an initial color HLS-segmentation focuses on regions likely to be traffic signs, using relaxed thresholds, at the cost of including many wrong candidate regions that must be discarded later. Otherwise the illumination conditions and the variations in signs color due to age and wear often leave signs out during the detection process.
- Afterward, a shape analysis tolerant to segmentation errors is carried out, again at the cost of including more wrong regions in the next stage. This is performed first by a filtering step according to general geometric restrictions, then a probabilistic adjustment of straight and circular segments into shapes [30]. A modified run-length encoding allows obtaining circular and straight segments supported by the external borders of the previously color-segmented blobs. A significant amount of circular segments is required to consider the blob as a candidate “prohibition” sign, and a significant amount of straight segments, aligned in the directions for triangles, is required to consider the blob as a candidate “danger” sign. Objects surviving this stage have a good likelihood of being traffic signs since they have the right colors and shapes.
- Finally, the next stage is about pattern matching, which is done over the inner region of candidate objects for the final decision. A standard grey scale normalized correlation stage is used, where patterns are manually obtained offline from sample signs. This approach proved to be insufficient, showing occasional mismatches between similar speed limit signs: namely 40, 60, 80 and 90 limitations. Therefore, one more *ad-hoc* discrimination procedure is applied, which is based on re-segmenting the speed limit text by dynamically adjusting a binarization threshold until the hole inside the ‘0’ digit is in the right proportion with respect to the contour of the same digit. At this point holes appearing in the tens digit (4, 6, 8, 9) are analyzed for the final discrimination.

**Figure 5.**
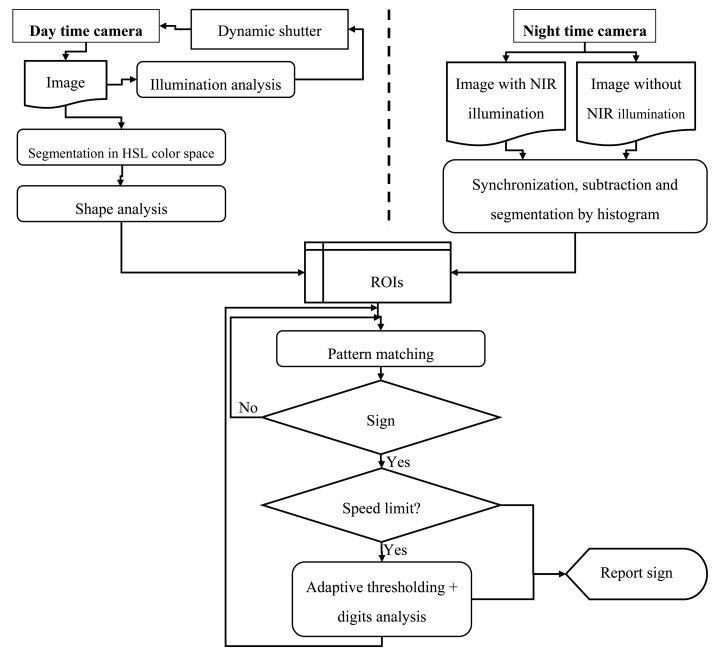
TDSR algorithm overview.

During nighttime, the use of a powerful near IR illuminator, which is simply a regular headlamp with a NIR filter aimed straight ahead, allows obtaining images where the reflective property of signs makes segmentation trivial. Removing the IR input filter in the camera, which is a conventional model with just a moderate gain in the IR spectrum, maximizes the IR light input to the camera sensor. Consequently, it allows the use of a very short integration time and avoids motion blur, reduces saturation of the inner region of the sign, and also dims other external light sources. Two time-close consecutive images, with and without NIR lighting, are taken. They are then combined by correcting the expected objects movement in the second shot, and then subtracting them. This allows discarding external light sources such as headlamps of incoming vehicles and keeping only highly reflecting objects. Often obtained image contains only signs, and sometimes other fortuitous reflecting objects. Then segmentation is straightforward, even leading to better results than during daytime. Since the image histogram is strongly bimodal, segmentation is performed by dynamic thresholding [31]. For each segmented image previously obtained, contours of the white regions are extracted. Once ROI are extracted, the subsequent sign reading steps are the same as those applied for daytime processing. The directed NIR illumination also reduces the chance of picking signs that do not apply to the driving lane, although that issue is not fully solved yet. Figure 5 shows a simplified diagram showing the steps in the TSDR algorithm.

### 3.2. Traffic Violations Recorder

The traffic violations recorder (TVR) is a module that depends on several parts: that is, the TSDR module, the EDR unit, and a number of other instruments. At present, the TVR module is focused on detecting only three traffic violations: namely “speed limit”, “stop sign”, and “forbidden turning”.

**Figure 6.**
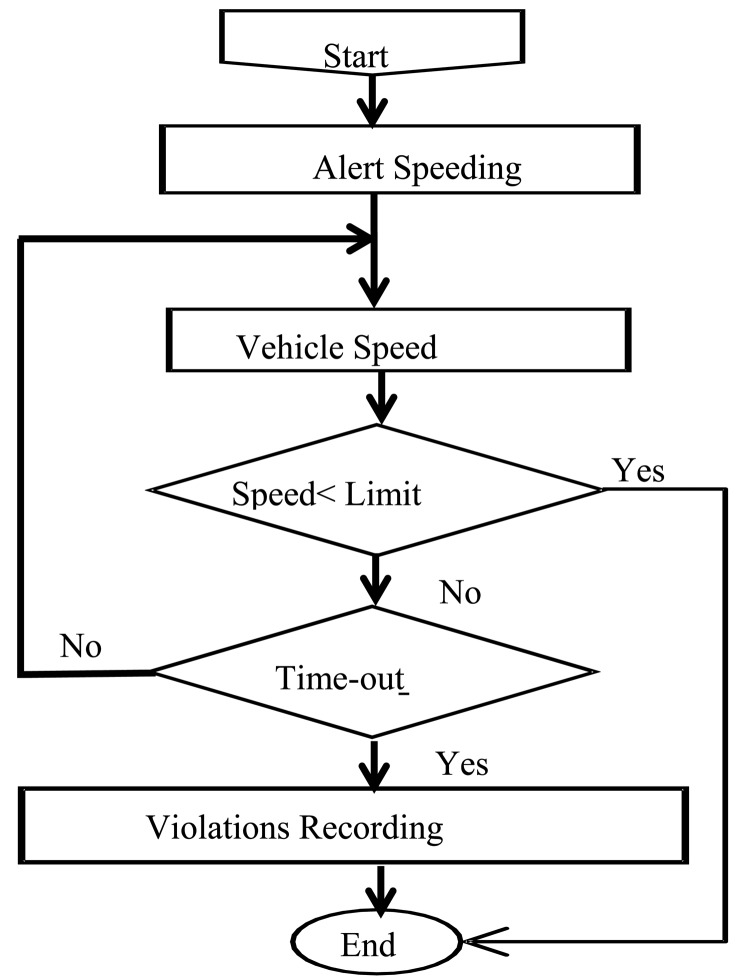
Flow diagram for handling speeding violation.

From the implementation point of view, each case has its own specific policy. For example, the “speed limit” violation is handled by comparing the actual vehicle speed, obtained from the ECU unit, with the detected speed limit. The issued alert is maintained during a predefined time-out period before recording the traffic sign violation if the driver does not react. The flow diagram used to manage “speed limit” is illustrated in Figure 6. In the same way, “stop sign” violation is managed by checking whether the vehicle speed has fallen to zero or not. Finally “forbidden turning” violation is handled by comparing the driver's intended direction, computed on the basis of the steering wheel angle and the vehicle's speed.

When a traffic violation is committed, its corresponding scenario is then recorded. This scenario is a set of data composed mainly by the type of the detected traffic sign, its GPS location, a picture of the surroundings, the vehicle speed, *etc.* The collected data is organized in the database unit according to drivers identified by their smart cards, and to trips associated to a calendar.

**Figure 7.**
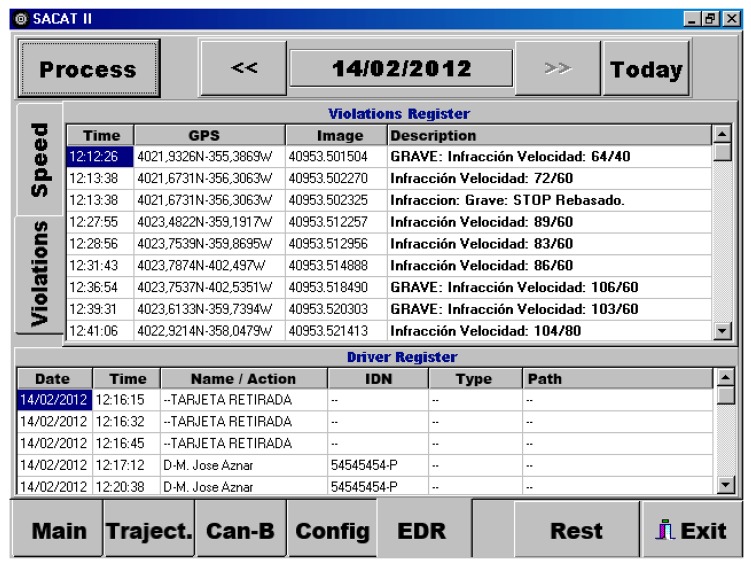
Snapshot of traffic violation register.

Drivers can also be provided with their traffic violations record allowing them to retrieve their own data, and therefore, making a self-diagnosis of their driving behavior. Figure 7 shows a snapshot of a tool implemented to retrieve traffic violation record committed by a driver.

**Figure 8.**
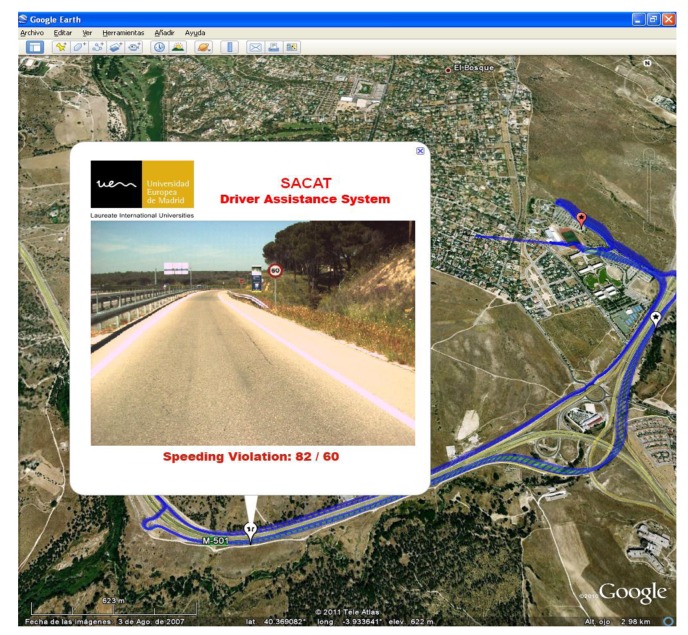
Snapshot of a Google Earth view showing an example of over speeding violation.

Another way to analyze driver's behavior is by generating a geographical map, allowing drivers to explore and visualize spatial and temporal information associated with a given trip using the standard Google Earth Map. In this aspect, a specific graphical tool has been developed, which gives divers a precise idea of the vehicle path, its speed, and marks the GPS locations of the recorded traffic violations. An example of such visualization is shown in Figure 8. By using this tool, drivers can retrieve hidden data such as images of the surroundings where a traffic violation have been committed by just moving the mouse around the map. A future application could also consider updating vehicles' navigator databases with repeatedly detected signs, or frequent sign violation points, since GPS position are already available.

As far as software development is concerned, the C++Builder Rapid Application Development is used, and computer vision issues are handled using the Matrox Imaging Library (MIL). MIL is a comprehensive set of optimized functions for developing machine vision and imaging software applications, and they are hardware-independent, meaning that a developer does not require an in-depth knowledge of the underlying hardware, and it includes tools for every step in image processing using C, C++, or C# programming languages. However, the current software includes a complex graphical user interface (GUI) for testing purposes, performs several analysis and test mechanisms, and it runs in a mono-processor mode, which naturally degrades overall performance. Even though, the system is able to process up to four frames per second (4 fps). In a future version, it will be considered to improve the frame rate by upgrading the hardware, optimizing the software algorithms, and enhancing detection performance for example by using statistics on several detections of the same sign.

On the other hand, for GPS location and geographical map representation, raw data is translated to keyhole markup language (KML) file used for modeling geographic features such as points, lines, images, polygons, and models for display in Google Earth. Like HTML, KML has a tag-based structure with names and attributes used for specific display purposes, and a KML file is processed by Google Earth in a similar way as HTML or XML files are processed by web browsers.

## 4. Discussion

The traffic sign detection and recognition module is capable of processing up to four frames per second (fps), so at normal speed (*i.e.*, 100 km/h), the system offers at least two opportunities to identify vertical signs. More identification opportunities are possible at lower speeds. The system is fully operational and works in real-time mode during daytime. The system has been tested under different daytime conditions, such as sunny, cloudy or rainy, in several driving runs including highways as well as urban areas at normal driving speeds. It is also tested in a night driving scenario. A total of 2000 kilometers of tests have been performed. The “true positive” detection rate reaches 90% of relevant signs in clear weather conditions, and it performs even better under cloudy conditions since scene illumination is softer and more homogenous. The night vision module makes segmentation easier so results are also better.

Evaluation is done in a per image basis along driving runs that typically take about 30–40 min. The amount of images taken is several thousand per driving run. When a new image contains a sign that should be read by the system, that is, it is a trained sign and it is within the detection range, the image is manually marked and stored. This action allows estimating the system performance in an off-line way by classifying the obtained results taking into account that detected signs are automatically stored. For example, in the reported daytime and clear weather drive test run, a total of 3122 images were taken. 250 of them are with signs within detection range, where 223 are “true positives”. Other 23 are “true negatives”, not reported by the system in the sense that are images with signs not trained or not applying to the driving lane. The remaining four signs were “false positives”, because they are detected but erroneously classified or they do not apply to the driving lane.

The remaining 2872 images, that did not include any traffic sign, and did not trigger any detection in the system, could be considered as “true negatives”. However, as this number is very high compared to the number of images having signs, including those results in the performance statistics, would greatly reduce the remaining detection rate. Therefore, only images with signs are included in the statistics. It is worth it to mention that discarding detected signs which do not apply to the driving lane is still an issue. Special efforts has been devoted to properly classify speed limit signs since the chance of mistakes between similar numbers (40, 60, 80, 90) are relevant. By further post-processing speed limit detections as previously described, the system has not committed speed limit signs mistakes since then.

The rate does not depend on the type of road (highway or urban road). However, the aforementioned rate falls slightly during rainy conditions. There are many other negative factors such as strong shadows caused by trees, intense fog, low contrast of vertical signs due to extreme lighting conditions, such as sun's position in front of the camera, or temporal out-of-shot signs when the vehicle is over the brow of a hill or in a very step turn.

The experiments performed for night vision are conducted in an off-line mode using a record of 1800 images collected over a number of tests in approximately 3-h driving, about 200 of those images corresponding to true traffic signs. The detection rate is nearly 92%, slightly better than the daytime score. False positive detection rate is less than 1%, and are mainly caused by objects that have similar shapes to some traffic signs. Table 1 summarizes the TSDR assessment in different conditions.

The figures shown in Table 1 correspond to just one typical driving test run. Many similar trips have been done along several years of work until getting those results. As far as the traffic violation recorder is concerned, the time elapsed from sign recognition and the issue of the alerts before registering the traffic violation obeys different policies depending on the traffic signs. In the case of speeding, the actual vehicle velocity, obtained from the CAN bus is constantly compared to the identified “Speed Limit” sign. When driving at 100 km/h, as the speed limit sign is detected at (50 or 30 m), an alert is issued and then the system grants to driver an extra time of about (2 to 1 s). In the case of “Stop Sign” and “Forbidden turning”, which normally take place within urban areas, the typical driving speed is now about 50–70 km/h or less. In this case, drivers have a reaction time of about 3 to 4 s. In all cases, warnings can be issued with sufficient notice to provide the driver with enough time to react to the on-coming traffic situation. Giving drivers early information permits the possibility of directing their attention to the most important traffic situations.

## 5. Conclusions

In this paper an experimental platform for recording traffic violations has been presented. The system is aimed at assisting drivers, and more particularly at reminding them of the presence of some specific traffic signs on the road. The warnings come in the form of acoustical messages emitted through the vehicle loudspeakers, and they are issued with sufficient time to provide the driver with enough notice to react to the oncoming traffic situation. Despite the alert and allowed reaction time, if a traffic violation is finally committed, it is recorded. The violation record consists of indicating the type of traffic sign, its GPS location, an image of the surroundings, and the vehicle's speed. The traffic violations are limited to three types: “speed limit”, “stop sign”, and “forbidden turning” signs. More situations such as “parking violation”, “no passing zone”, and “red light”, are under consideration and development.

Drivers can be provided with their own traffic violation register as feedback, and can visualize the spatial and temporal information of their traffic violation register using the standard Google Earth map. This system may contribute to prevent drivers from committing traffic violations and infringements and can be used to persuade them to change their driving styles. Although it is far from being commercialized, the development presented in this paper offers real and promising framework for experimentation. Finally, it is also planned to carry out an experiment with the collaboration of the Spanish Traffic Agency to perform qualitative and quantitative studies about driver perception about the advantages of using a system warning them about their traffic violations.

## Figures and Tables

**Table 1.** Traffic Sign detection and recognition assessment.

	**Daytime and Clear Weather Sample Drive Run Including 250 Signs (3122 Total Images)**		**Daytime and Cloudy Weather Sample Drive Run Including 140 Signs (1816 Total Images)**		**Nighttime Sample Images Including 200 Signs (1800 Total Images)**	

	**Count**	**rate**	**count**	**rate**	**count**	**rate**
True positives	223	89.2%	127	90.7%	183	91.5%
True negatives	23	9.2%	11	7.9%	15	7.5%
False positives	4	1.6%	2	1.4%	2	1.0%

## References

[1] eCall Report (May 2014).

[2] Horrey W.J., Lesch M.F. (2009). Driver-Initiated Distractions: Examining Strategic Adaptation for In-Vehicle Task Initiation. Accid. Anal. Prev..

[3] Dong Y., Hu Z., Uchimura K., Murayama N. (2011). Driver Inattention Monitoring System for Intelligent Vehicles: A Review. IEEE Intell. Transp. Syst..

[4] Horrey W.J., Lesch M.F., Garabet A. (2008). Assessing the Awareness of Performance Decrements in Distracted Drivers. Accid. Anal. Prev..

[5] Eriksson M., Papanikolopoulos P.N. (2001). Driver Fatigue: A Vision-Based Approach to Automatic Diagnosis. Transp. Res. Part C Emerg. Technol..

[6] Bahlmann C., Ying Z., Ramesh V., Pellkofer M., Koehler T. A system for traffic sign detection, tracking, and recognition using color, shape, and motion information.

[7] McCall J.C., Trivedi M.M. (2006). Video-Based Lane Estimation and Tracking for Driver Assistance: Survey, System, and Evaluation. IEEE Intell. Transp. Syst..

[8] Manubhai M., Trivedi T.G., McCall J.C. (2007). Looking-In and Looking-Out of a Vehicle: Computer-Vision-Based Enhanced Vehicle Safety. IEEE Intell. Transp. Syst..

[9] Handmann U., Kalinke T., Tzomakas C., Wernerand M., Seelen W.V. (2000). An Image Processing System for Driver Assistance. Image Vis. Comput..

[10] Bergasa L.M., Nuevo J., Sotelo M.A., Barea R., Lopez M.E. (2006). Real-Time system for monitoring driver vigilance. IEEE Intell. Transp. Syst..

[11] Escalera A., Armingol J.M., Mata M. (2003). Traffic sign recognition and analysis for intelligent vehicles. Image Vis. Comput..

[12] Broggi A., Cerri P., Ghidoni S., Grisleri P., Jung H.G. (2009). A New Approach to Urban Pedestrian Detection for Automatic Braking. IEEE Intell. Transp. Syst..

[13] Jiménez F., Naranjo J.E., Gómez O. (2012). Autonomous manoeuvring systems for collision avoidance on single carriageway roads. Sensors.

[14] Keller C.G., Dang T., Fritz H., Joos A., Rabe C., Gavrila D.M. (2011). Active pedestrian safety by automatic braking and evasive steering. IEEE Intell. Transp. Syst..

[15] Dang T., Desens J., Franke U., Gavrila D., Schäfers L., Ziegler W. (2012). Steering and Evasion Assist. Handbook of Intelligent Vehicles.

[16] Takeda K., Miyajima C., Suzuki T., Kurumida K., Kuroyanagi Y., Ishikawa H. Improving Driving behavior by allowing drivers to browse their own recorded driving data.

[17] Perez A., Garcia M.I., Nieto M., Pedraza J.L., Rodriguez S., Zamorano J. (2010). Argos: An advanced in-vehicle data recorder on a massively sensorized vehicle for car driver behavior experimentation. IEEE Intell. Transp. Syst..

[18] Toledo T., Musicant O., Lotanc T. (2008). In-Vehicle data recorders for monitoring and feedback on drivers' behaviour. Transp. Res. Part C Emerg. Technol..

[19] Ma X., Andreasson I. (2007). Behavior measurement, analysis, and regime classification in car following. IEEE Intell. Transp. Syst..

[20] British Department for Transport: Report No. 122: “Road Safety Research”.

[21] Aliane N., Fernandez J., Bemposta S., Mata M. Traffic violation alert and management.

[22] Aliane N., Fernandez J., Bemposta S., Mata M. Driver behavior monitoring system based on traffic violation.

[23] Rajalin S. (1994). The connection between risky driving and involvement in fatal accidents. Accid. Anal. Prev..

[24] Fleyeh H. Color detection and segmentation for road and traffic signs.

[25] Escalera A., Armingol J.M., Pastor J.M., Rodríguez F.J. (2004). Visual sign information extraction and identification by deformable models for intelligent vehicles. IEEE Trans. Intell. Transp. Syst..

[26] Prieto M., Allen A. (2009). Using self-organizing maps in the detection and recognition of road signs. Image Vis. Comput..

[27] Chourasia J.N., Bajaj P. Centroid based detection algorithm for hybrid traffic sign recognition system.

[28] Gómez H., Maldonado S., Jiménez P.G., Gómez H., Lafuente-Arroyo S. (2010). Goal evaluation of segmentation for traffic sign recognition. IEEE Trans. Intell. Transp. Syst..

[29] Escalera S., Baró X., Pujol O., Vitria J., Radeva P. (2011). Traffic-Sign Recognition Systems (SpringerBriefs in Computer Science).

[30] Blake A., Isard M. (1998). Active Contours: The Application of Techniques from Graphics, Vision, Control Theory and Statistics to Visual Tracking of Shapes in Motion.

[31] Shapiro L.G., Stockman G.C. (2001). Computer Vision.

